# Syndrome of inappropriate antidiuretic hormone secretion following immune effector cell–associated neurotoxicity syndrome during blinatumomab therapy: a case report

**DOI:** 10.1186/s40780-026-00606-x

**Published:** 2026-07-10

**Authors:** Misato Yoshihara, Satoshi Yuyama, Osamu Yasumuro, Ryohkan Funakoshi

**Affiliations:** Department of Pharmacy, Kameda Medical Center, 929 Higashi-cho, Kamogawa-shi, Chiba 296-8602 Japan

**Keywords:** Blinatumomab, Syndrome of inappropriate antidiuretic hormone secretion, Hyponatremia, Immune effector cell–associated neurotoxicity syndrome, Interleukin-6

## Abstract

**Background:**

Blinatumomab (BLINA) causes immune-related toxicities, such as cytokine release syndrome and immune effector cell–associated neurotoxicity syndrome (ICANS). Although hyponatremia associated with interleukin (IL)-6–mediated vasopressin secretion has been reported with chimeric antigen receptor T-cell therapy, similar reports with bispecific antibodies are scarce.

**Case presentation:**

A woman in her 60s with B-cell acute lymphoblastic leukemia developed grade 3 ICANS on day 3 of the second BLINA cycle. On day 13, the patient experienced grade 4 hyponatremia (serum sodium, 106 mmol/L). Treatment with 3% saline and tolvaptan improved symptoms. No other causes, such as central nervous system disease, adrenal insufficiency, or thyroid dysfunction, were identified. Based on the clinical findings and the exclusion of other etiologies, BLINA-induced syndrome of inappropriate antidiuretic hormone secretion (SIADH) was diagnosed. After confirming a decrease in IL-6 levels, tolvaptan was tapered and discontinued, and no recurrence of hyponatremia was observed.

**Conclusions:**

BLINA may be involved in SIADH development, potentially via increased central IL-6 levels following ICANS treatment, which may promote arginine vasopressin secretion. In patients who develop ICANS during BLINA therapy, close monitoring of serum sodium levels may be a useful part of neurological assessment. If hyponatremia occurs, clinicians should promptly consider SIADH and initiate the appropriate management.

## Background

Blinatumomab (BLINA) exerts its antitumor effects by bridging CD3 on cytotoxic T cells and CD19-positive B-cell tumors, thereby activating T cells. This process involves the transient release of inflammatory cytokines, leading to characteristic adverse effects, such as cytokine release syndrome (CRS) and immune effector cell–associated neurotoxicity syndrome (ICANS). This adverse effect profile resembles that of chimeric antigen receptor (CAR) T-cell therapy. An association between ICANS and hyponatremia has been reported in CAR T-cell therapy [1], suggesting that elevated interleukin-6 (IL-6) levels may cause inappropriate arginine vasopressin (AVP) secretion [2, 3]. However, to our knowledge, no such association has been reported for bispecific antibodies. We report a case of syndrome of inappropriate antidiuretic hormone secretion (SIADH) following ICANS induced by BLINA.

## Case presentation

A 60-year-old woman presented to our emergency department with petechiae and thrombocytopenia. She was diagnosed with B-cell acute lymphoblastic leukemia (B-ALL). Owing to minor *BCR-ABL1* positivity, she was classified as having Philadelphia chromosome–positive B-ALL and was initiated on hyper-fractionated cyclophosphamide, vincristine, doxorubicin, and dexamethasone (hyper-CVAD) plus ponatinib (30 mg for 14 days) combination therapy. After the first cycle of therapy, the patient developed atrial fibrillation and polymorphic ventricular tachycardia. In addition, given the *BCR-ABL1* positivity, the treatment regimen was modified to BLINA combined with dasatinib on the next cycle. In the timeline of this case, day X means day 1 of the second cycle of BLINA.

Three months before day X, she achieved molecular complete remission. The patient’s medical history included thoracoabdominal aortic aneurysm, aortic valve insufficiency, combined valvular disease, atrial fibrillation, polymorphic ventricular tachycardia, hypertension, adenomatous goiter, and allergic rhinitis. Her medication comprised levetiracetam 1000 mg/day, spironolactone 50 mg/day, lemborexant 2.5 mg/day, carvedilol 20 mg/day, sacubitril/valsartan 50 mg/day, acyclovir 200 mg/day, esomeprazole 10 mg/day, edoxaban 30 mg/day, and bilastine 20 mg/day. She had no history of allergies. Adverse reactions included rash with amoxicillin, trimethoprim-sulfamethoxazole, and dasatinib, and suspected destructive thyrotoxicosis with amiodarone.

Forty-seven days before day X, we initiated the first cycle of BLINA therapy and completed its administration without CRS or ICANS. On day X, we initiated a second cycle of BLINA 28 µg/day plus dasatinib 140 mg/day combination therapy.

**Fig. 1:**
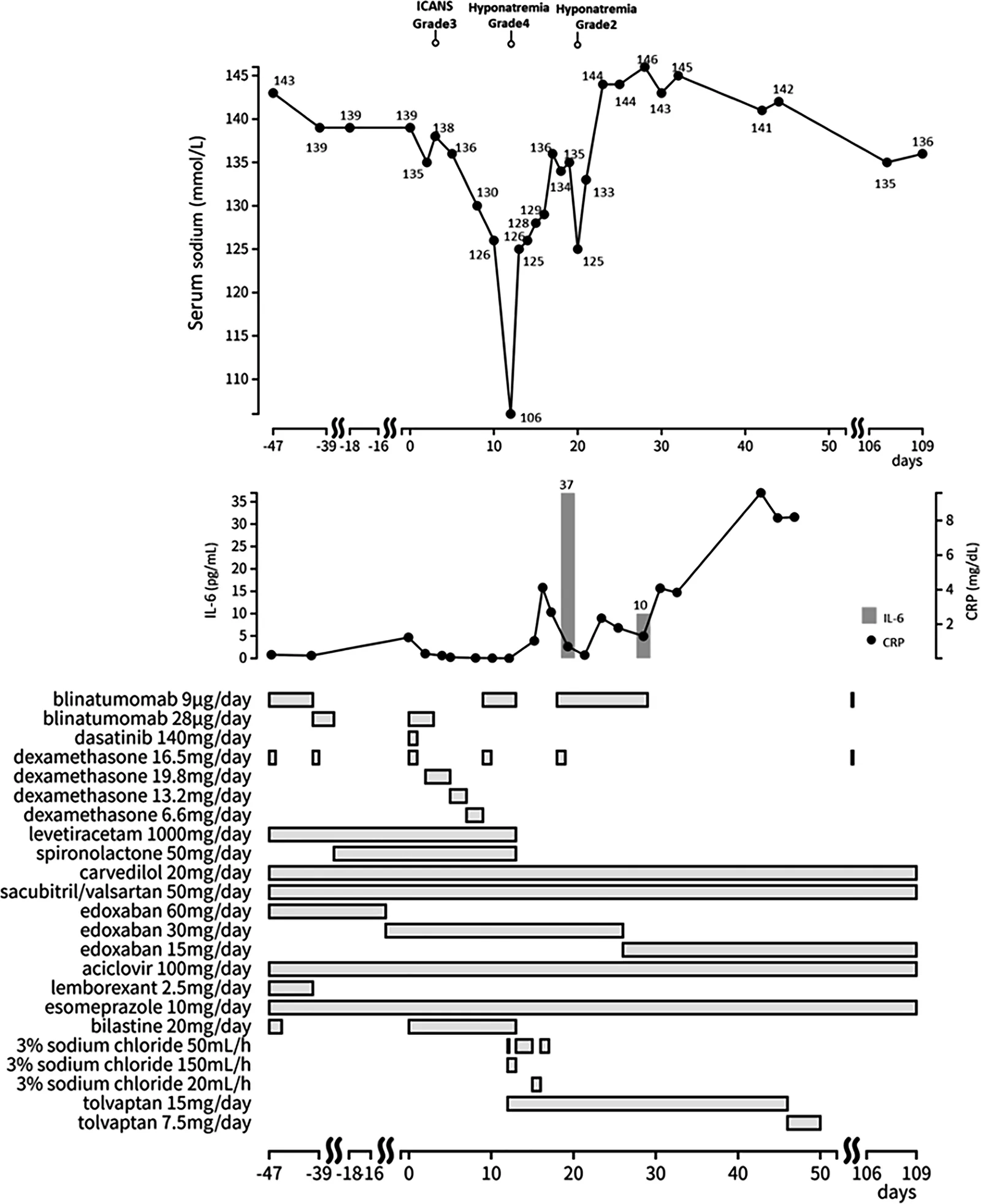
Clinical course during second cycle of BLINA therapy. Day X corresponds to day 1 of the cycle. The x-axis indicates the number of days since day X. ICANS episodes occurred on day X + 2; the first and second hyponatremia episodes occurred on days X + 12 and X + 20, respectively. For the treatment of hyponatremia, 3% NaCl continuous infusion was administered from day X + 12, but it did not yield sufficient effect; thus, tolvaptan 15 mg/day was initiated. Sodium levels recovered, and BLINA was restarted on day X + 18. Despite tolvaptan administration, grade 2 asymptomatic hyponatremia (sodium, 125 mmol/L) was observed on day X + 20, but it subsequently resolved spontaneously. BLINA, blinatumomab; ICANS, immune effector cell-associated neurotoxicity syndrome

On day X + 1, the patient developed a rash. Dasatinib administration was discontinued because of suspected drug-related complications. On day X + 2, global aphasia was noted (hospital name and date replaced with meaningless words) and grade 3 ICANS was diagnosed based on the American Society for Transplantation and Cellular Therapy (ASTCT) consensus grading. BLINA was interrupted, and dexamethasone 19.8 mg/day was administered. On day X + 3, aphasia resolved, and the Immune Effector Cell Encephalopathy score recovered to 10 points (maximum score: 10). Dexamethasone was tapered and administered until day X + 8. On day X + 9, BLINA was restarted at a reduced dose of 9 µg/day, with dexamethasone 16.5 mg as premedication, because the patient had developed grade 3 ICANS, in accordance with the prescribing information. On day X + 12, grade 4 asymptomatic hyponatremia (sodium, 106 mmol/L) was noted. The patient’s blood pressure was 99/48 mmHg, and her pulse was 79 bpm, with no signs of dehydration. Other findings included hypo-osmolarity, hyperosmotic urine, elevated urinary sodium, and elevated AVP concentration, leading to a diagnosis of SIADH. BLINA administration was then discontinued, and continuous infusion of 3% NaCl was initiated. The blood and urine test findings on day X + 12 are presented in Table 1. A 3% NaCl continuous infusion was administered but did not yield sufficient effect; thus, tolvaptan 15 mg/day was initiated. As the sodium level recovered, BLINA was restarted on day X + 18 with 16.5 mg of dexamethasone premedication. Despite tolvaptan administration, grade 2 asymptomatic hyponatremia (sodium, 125 mmol/L) was observed on day X + 20, but it resolved spontaneously. BLINA administration was completed on day X + 27. IL-6 (reference range < 7 pg/mL) was elevated at 37 pg/mL on day X + 19 but decreased to 10 pg/mL by day X + 28. Tolvaptan was therefore tapered and discontinued, with no recurrence of hyponatremia (Fig. 1). The CRS and IL-6 levels are presented in Fig. 1.

**Table 1:** Blood tests and urine tests

Investigation	Laboratory values		Normal range
	X+12^a^	X+20^b^	
Serum sodium (mmol/L)	106	125	136–143
Plasma osmolality (mOsm/kg H_2_O)	235	−	270–290
Urine osmolality (mOsm/kg H_2_O)	525	−	50–1300
Urinary sodium (mmol/L)	125.1	−	−
plasma vasopressin (pg/mL)	5.5	−	0.0–2.8
Cortisol (µg/dL)	13.9	−	7.07–19.6
ACTH (pg/mL)	11.7	−	8.7–61.5
TSH (µIU/mL)	0.041	−	0.50–5.00
Free T4 (ng/dL)	2.69	−	0.90–1.70
Hematocrit value (%)	17.4	−	35.1–44.4
Urea nitrogen (mg/dL)	26	23	8.0–20.0
Serum creatinine (mg/dL)	0.23	0.28	0.46–0.79
Serum albumin (g/dL)	2.4	-	4.1–5.1
BNP (pg/mL)	65.5	-	0–18.4

^a^First hyponatremia episode on day 12

^b^Second hyponatremia episode on day 20

X, day 1 of the second cycle of blinatumomab therapy; ACTH, adrenocorticotropic hormone; TSH, thyroid-stimulating hormone; free T4, free thyroxine; BNP, B-type natriuretic peptide

## Discussion and conclusions

In the present case, ICANS developed during the administration of BLINA, followed by severe hyponatremia and typical SIADH. Laboratory findings consistent with SIADH included hypo-osmolar blood, hyperosmotic urine, elevated urinary sodium, and elevated serum AVP [4]. Given the lack of signs of dehydration, fluid volume was considered normal.

SIADH is the most common cause of hyponatremia. SIADH is a condition in which excessive secretion of antidiuretic hormone (vasopressin) promotes water reabsorption in the renal collecting tubules, leading to dilutional hyponatremia. Clinical symptoms may include central nervous system (CNS) symptoms such as cerebral edema, headache, nausea/vomiting, and somnolence. A study of hospitalized patients with serum sodium concentrations of 125 mmol/L showed an overall mortality rate of 27%, which is significantly higher than the 9% rate in the normal sodium group (*P* = 0.009) [5]. A previous study investigating the causes of SIADH in 555 hospitalized patients diagnosed with the condition reported the following distributions: idiopathic (15.9%), drug-induced (26.5%), pulmonary disease (12.3%), CNS disease (7.2%), malignancy (27.7%), and pain or nausea (10.4%) [6]. Representative causative drugs for drug-induced SIADH include carbamazepine, valproic acid, amitriptyline, imipramine, selective serotonin reuptake inhibitors (such as paroxetine and sertraline), haloperidol, chlorpromazine, cyclophosphamide, cisplatin, and vincristine [7].

Endocrine disorders critical for differentiating SIADH, such as adrenal insufficiency and hypothyroidism, were also ruled out, and renal function was preserved. Furthermore, complete molecular remission was achieved at 3 months before day X, and no CNS involvement was detected, suggesting that B-ALL was an unlikely cause of SIADH in our patient.

In this case, vincristine, which was administered 4 months before day X, was the representative causative agent of SIADH. Vincristine is thought to exhibit neurotoxicity in the hypothalamic-pituitary axis, altering the normal osmotic control of antidiuretic hormone secretion [8]. However, in a previous report, cases of SIADH were diagnosed based on symptomatic hyponatremia developing within 4–10 days after vincristine administration [9]. This temporal correlation was not present in our case, and no significant hyponatremia was observed during or after hyper-CVAD therapy. Another causative agent is the anticonvulsant levetiracetam, which causes SIADH. However, in this case, levetiracetam was administered prophylactically starting from the first course of BLINA [10], and no significant hyponatremia was observed during this period. Furthermore, the timing did not align with the reported onset period for SIADH associated with levetiracetam (days 4–7 after initiation) [11, 12]. Therefore, vincristine and levetiracetam were considered unlikely to be the causative agents. Although dasatinib has been reported to cause SIADH [13], in the present case, the temporal relationship was not consistent, as severe hyponatremia developed after its discontinuation. Furthermore, although dasatinib was readministered on day X + 136, no recurrence of hyponatremia was observed, suggesting that dasatinib was unlikely to be the causative agent. Spironolactone and sacubitril/valsartan cause hyponatremia by promoting urinary sodium excretion. However, no signs of dehydration were observed, and despite the continuous prior administration of these agents, no previous episodes of hyponatremia were observed.

Dexamethasone-related hypovolemic hyponatremia has been reported [14]. In contrast, the present case showed no evidence of volume depletion and was consistent with euvolemic hyponatremia owing to SIADH, making dexamethasone an unlikely cause. Although dexamethasone was administered as premedication in the first cycle, hyponatremia was not observed. Furthermore, although dilutional hyponatremia owing to worsening heart failure was considered possible, no worsening of the B-type natriuretic peptide levels was observed. Based on these findings, alternative causes other than BLINA, such as drug-induced, endocrine, renal, or tumor infiltration hyponatremia, were deemed unlikely.

Elevated IL-6 levels peaked within 24 h of BLINA administration and rapidly declined within 48 h; no such elevation was observed again after the first cycle [15]. Despite being in the second cycle, the patient showed an IL-6 level of 37 pg/mL at day X + 19, which was higher than the reference value (< 7 pg/mL). By day X + 28, the IL-6 level decreased to 10 pg/mL, and no recurrence of hyponatremia was observed, even after discontinuation of tolvaptan.

Studies on CAR T-cell therapy have reported an inverse correlation between IL-6 elevation and hyponatremia, suggesting that increased IL-6 levels promote AVP secretion and contribute to SIADH development [2]. The median time to the onset of hyponatremia following CAR T-cell therapy has been reported to be 5 days (range, 1–32 days) [2]. To our knowledge, there are no reports of SIADH induced by BLINA, a bispecific antibody; however, given the similar adverse event profiles, it is possible that BLINA could induce SIADH associated with elevated IL-6 levels. ICANS has been shown to be associated with blood–brain barrier (BBB) disruption [16]. We hypothesize that BLINA-associated ICANS may increase BBB permeability, allowing peripheral IL-6 to enter the CNS and be associated with SIADH. This case was assessed as probable (total score, 5–8) using the Naranjo Adverse Drug Reaction Probability Scale [17] (Table 2).

**Table 2:** Calculation of the probability of an adverse drug reaction occurring in the patient based on the Naranjo Adverse Drug Reaction Probability Scale

No.		Yes	No	Unknown	Score
1	Are there previous conclusive reports on this reaction?	+ 1	0	0	0
2	Did the adverse event appear after the suspected drug was administered?	+ 2	−1	0	+ 2
3	Did the adverse reaction improve when the drug was discontinued or a specific antagonist was administered?	+ 1	0	0	+ 1
4	Did the adverse reaction reappear when the drug was readministered?	+ 2	−1	0	+ 2
5	Are there alternative causes (other than the drug) that could on their own have caused the reaction?	−1	+ 2	0	+ 2
6	Did the reaction reappear when a placebo was given?	−1	+ 1	0	0
7	Was the drug detected in any bodily fluid in toxic concentrations?	+ 1	0	0	0
8	Was the reaction more severe when the dose was increased, or less severe when the dose was decreased?	+ 1	0	0	0
9	Did the patient have a similar reaction to the same or similar drugs in any previous exposure?	+ 1	0	0	0
10	Was the adverse event confirmed by any objective evidence?	+ 1	0	0	+ 1
		Total score			**8**

This case report has several limitations. First, the IL-6 levels in the peripheral blood and cerebrospinal fluid were not measured during ICANS or at the onset of severe hyponatremia. Although we speculated the possibility of central IL-6 elevation after ICANS, we could not directly prove an IL-6 increase during ICANS or severe hyponatremia. The IL-6 peak is short-lived, appearing only during the first 2 days of the first cycle and not recurring in subsequent cycles [15]. However, despite being in the second cycle, IL-6 levels remained elevated the day after BLINA restarted, followed by the recurrence of hyponatremia. After the IL-6 level decreased, discontinuation of tolvaptan did not result in the recurrence of hyponatremia. Second, There was no CRS or a marked increase in C-reactive protein (CRP) levels, which are thought to correlate with IL-6 elevation. ICANS without CRS complications has been reported. We hypothesize that central IL-6 elevation may not necessarily be accompanied by peripheral CRP elevation. Third, although the Naranjo Adverse Drug Reaction Probability Scale rated this case as probable, we hypothesize that the hyponatremia resulted from a secondary mechanism mediated by increased BBB permeability and elevated IL-6 levels rather than a direct mechanism caused by BLINA. Therefore, the Naranjo Adverse Drug Reaction Probability Scale may have limitations in this case, as the mechanism is likely indirect and mediated by ICANS rather than a direct effect of BLINA; thus, the findings should be interpreted with caution. Nevertheless, considering the temporal association in this case, the exclusion of other causes, the recurrence upon readministration, and the existence of previously reported cases with similar adverse event profiles in CAR T-cell therapy, BLINA may be associated with the onset of SIADH. The mechanism underlying SIADH in this case may involve multiple steps. First, previous studies have suggested that elevated IL-6 levels can stimulate AVP secretion, contributing to the development of SIADH [2, 3]. Second, ICANS has been associated with disruption of the BBB [16, 18], which may allow peripheral cytokines, including IL-6, to influence central regulatory pathways. However, direct evidence linking ICANS-associated blood–brain barrier disruption to increased central IL-6 levels remains limited. Therefore, although these findings suggest a possible mechanism, the relationship among ICANS, IL-6 elevation, and SIADH should be interpreted with caution. Compared with CAR T-cell therapy, the lower incidence of ICANS with bispecific antibodies may explain why few cases have reported an association with severe hyponatremia like this one.

Although the precise reason why ICANS and subsequent SIADH developed during the second cycle but not during the first cycle remains unclear, one possible explanation is immune priming. The initial cycle of BLINA may have enhanced T-cell activation and driven the expansion of effector memory T cells, leading to a more robust cytokine response upon re-exposure during the second cycle. Previous studies have also demonstrated dynamic changes in peripheral T-cell kinetics following BLINA treatment [15]. In addition, cases of ICANS occurring during later cycles have been reported [19], suggesting that immune-related toxicity may develop beyond the first cycle.

In cases where ICANS develops during BLINA administration, strict monitoring of serum sodium levels may be a useful part of neurological assessment. Prompt intervention that considers the possibility of SIADH is necessary if hyponatremia is detected. This case report represents a rare instance of SIADH that is temporally associated with ICANS during BLINA administration. Further studies are required to elucidate the underlying mechanisms.

## Data Availability

No datasets were generated or analysed during the current study.

## Abbreviations

AVP: Arginine vasopressin
B-ALL: B-cell acute lymphoblastic leukemia
BBB: Blood–brain barrier
BLINA: Blinatumomab
CAR T cell: Chimeric antigen receptor T cell
CNS: Central nervous system
CRP: C-reactive protein
CRS: Cytokine release syndrome
Hyper-CVAD: Hyper-fractionated cyclophosphamide, vincristine, doxorubicin, and dexamethasone
ICANS: Immune effector cell–associated neurotoxicity syndrome
IL-6: Interleukin-6
SIADH: Syndrome of inappropriate antidiuretic hormone secretion
